# Dexamethasone versus ondansetron in the prevention of postoperative nausea and vomiting in patients undergoing laparoscopic surgery: a meta-analysis of randomized controlled trials

**DOI:** 10.1186/s12871-015-0100-2

**Published:** 2015-08-15

**Authors:** Xian-Xue Wang, Quan Zhou, Dao-Bo Pan, Hui-Wei Deng, Ai-Guo Zhou, Fu-Rong Huang, Hua-Jing Guo

**Affiliations:** 1 Jiangsu Province Key Laboratory of Anesthesiology, Xuzhou Medical College, Xuzhou, Jiangsu China; 2 Jiangsu Province Key Laboratory of Anesthesia and Analgesia Application Technology, Xuzhou, Jiangsu China; 3 Science & Education Division of the First People’s Hospital of Changde City, Changde, Hunan China; 4 Department of Anesthesiology of the First People’s Hospital of Changde City, Changde, Hunan China

## Abstract

**Background:**

Dexamethasone is an antiemetic alternative to ondansetron. We aimed to compare the effects of dexamethasone and ondansetron in preventing postoperative nausea and vomiting (PONV) in patients undergoing laparoscopic surgery.

**Methods:**

We searched PubMed, Embase, Medline and Cochrane Library (from inception to July 2014) for eligible studies. The primary outcome was the incidence of PONV during the first 24 h after surgery. The secondary outcomes included PONV in the early postoperative stage (0–6 h), PONV in the late postoperative stage (6–24 h), and the postoperative anti-emetics used at both stages. We calculated pooled risk ratios (RR) and 95 % CIs using random- and fixed-effects models.

**Results:**

Seven trials involving 608 patients were included in this meta-analysis, which found that dexamethasone had a comparable effectiveness in preventing PONV (RR, 0.91; 95 % CI, 0.73-1.13; *P* = 0.39) with that of ondansetron within 24 h of laparoscopic surgery, with no evidence of heterogeneity among the studies (I^2^ = 0 %; *P* = 0.71). In the early postoperative stage (0–6 h), ondansetron was better at decreasing PONV than dexamethasone (RR, 1.71; 95 % CI, 1.05-2.77; *P* = 0.03), while in the late postoperative stage (6–24 h), dexamethasone was more effective in preventing PONV than ondansetron (RR, 0.51; 95 % CI, 0.27-0.93; *P* = 0.03). There was no significant difference in the postoperative anti-emetics used (RR, 0.90; 95 % CI, 0.67-1.19; *P* = 0.45).

**Conclusions:**

Dexamethasone was as effective and as safe as ondansetron in preventing PONV. Dexamethasone should be encouraged as an alternative to ondansetron for preventing PONV in patients undergoing laparoscopic surgery.

## Background

Postoperative nausea and vomiting (PONV) is a common distressing symptom in patients undergoing laparoscopic surgery and can contribute to anxiety, dehydration, metabolic abnormality, wound disruption, delayed recovery and other issues [1]. The incidence of PONV varies from 20 to 80 %, and it is an economic and social burden. Ondansetron is a selective 5-HT_3_ receptor antagonist, that exhibits an anti-emetic action by antagonizing vomiting signals in the afferent pathway from the stomach or small intestine and solitary tract nucleus, and is effective at preventing PONV [2, 3], however the high cost of this drug has prevented it from being widely used. Dexamethasone, a corticosteroid, was first reported as an effective anti-emetic agent in patients undergoing cancer chemotherapy in 1981 [4, 5]. Wang et al. [6] confirmed that dexamethasone is most effective when it is administered at the induction rather than at the termination of anaesthesia. However, the mechanism underlying the anti-emetic effects of dexamethasone is still unknown. It may be involved in central inhibition of prostaglandin synthesis, or it may cause a decrease in serotonin turnover in the central nervous system [5, 7, 8].

Today, cost-benefit analyses have become an important factor when considering what drugs to use as prophylactic antiemetics. The cost-to-benefit ratio for the patient was much higher in the ondansetron group than in the dexamethasone group [9]. However, it has not been established whether dexamethasone is a cost-effective alternative to ondansetron in the prevention of PONV in patients undergoing laparoscopic surgery. It is highly necessary to conduct a meta-analysis to assess the results of the currently published studies on this topic. Therefore, we performed a systematic review to compare the efficacy of dexamethasone with that of ondansetron.

## Methods

This systematic review was carried out according to the guidelines of the preferred reporting items for systematic reviews and meta-analyses (PRISMA) [10]. We prospectively registered our system review at PROSPERO. (Registration number: CRD420140013064).

### Data sources and search strategy

PubMed, Embase, Medline and Cochrane Library databases were searched from inception to July, 2014 for relevant studies that investigated the differences in the anti-emetic effects of dexamethasone and ondansetron. The following search terms were used: Dexamethasone, Hexadecadrol, Methylfluorprednisolone, Decameth, “Foy Brand of Dexamethasone”, Decaspray, “Merck Brand of Dexamethasone”, Dexasone, “ICN Brand of Dexamethasone”, Dexpak, “ECR Brand of Dexamethasone”, Maxidex, “Alcon Brand of Dexamethasone”, Millicorten, Oradexon, Decaject, “Merz Brand 1 of Dexamethasone”, “Decaject L.A.”, “Decaject-L.A.”, “Merz Brand 2 of Dexamethasone”, Hexadrol, Ondansetron, “Ondansetron, (+,-)-Isomer”, “Ondansetron, (R)-Isomer”, “Ondansetron, (S)-Isomer”, “GR-38032F”, “GR 38032F”, “GR 38032F”, Zofran, “Ondansetron Hydrochloride”, “Hydrochloride, Ondansetron”, “Ondansetron Monohydrochloride Dihydrate”, “Dihydrate, Ondansetron Monohydrochloride”, “Monohydrochloride Dihydrate, Ondansetron”, “Ondansetron Monohydrochloride”, “Monohydrochloride, Ondansetron”, SN 307, “SN-307”, “Laparoscopy”, Laparoscopies, Peritoneoscopy, Peritoneoscopies, Celioscopy, Celioscopies, “Surgical Procedures, Laparoscopic”, “Laparoscopic Surgical Procedure”, “Surgical Procedure, Laparoscopic”, “Procedure, Laparoscopic Surgical”, “Procedures, Laparoscopic Surgical”, “Surgery, Laparoscopic”, “Laparoscopic Surgery”, “Laparoscopic Surgeries”, “Surgeries, Laparoscopic”, “Laparoscopic Surgical Procedures”, PONV, vomiting, emesis and nausea. A manual search of the reference sections of the included trials, published meta-analyses, and pertinent review articles was also conducted to identify additional relevant articles. If duplicated data were presented in several publications, only the most recent, largest or most complete study was included in this meta-analysis.

### Study selection

The original studies included in this meta-analysis were based on PICOS (patient, intervention, comparison, outcomes and study design) as follows: (a) P: American Society of Anesthesiology (ASA) I/II grade adult patients undergoing laparoscopic surgery; (b) I and C: dexamethasone and ondansetron respectively; (c) O: reporting the incidence of PONV; (d) S: only randomized controlled trials (RCTs). Only articles published in English were included. Patients who had a history of nausea or vomiting or who had been given H_2_ blockers 48 h prior to the operation were excluded. Patients with the following characteristics were also excluded: history of motion sickness, facing kidney problems with a high level of BUN or Cr, history of allergy to the study drug, body mass index (BMI) > 35 and being pregnant or menstruating.

### Data extraction

The characteristics of the patients (number of patients, ASA rating, age, gender, type of surgery and anaesthesia) and the trial designs (intervention, follow-up duration and reported outcomes) were also recorded. If the data mentioned above were unavailable in the article, the corresponding authors were called upon to obtain the missing information. All data were independently extracted using a standard data collection form by 2 reviewers (XXW and FRH), then, the collected data were checked and entered into Review Manager analysis software (RevMan) Version 5.3. All discrepancies were reviewed and a consensus was reached by discussion with a third author (DBP). The reasons that studies were excluded were recorded.

### Assessment of study quality

A critical evaluation of the quality of the included studies was performed by two reviewers (XXW and HJG) using a 5-point Jadad scale [11]. The main categories consisted of the following five items: “Was the study described as randomized?”, “Was the method used to obtain the sequence of randomization described and appropriate (random numbers, computer-generated, etc.)?”, “Was the study described as double-blind?”, “Was the method of double-blinding described and appropriate (identical placebo, active placebo, dummy, etc.)?”, and “Was there a description of withdrawals and drop-outs?”. Scores of four and five indicated a high methodological quality.

### Assessment of risk of bias

Two reviewers (XXW and QZ) independently evaluated the risk of bias according to the recommendations of the Cochrane Collaboration [12]. The principal categories consisted of random sequence generation, allocation concealment, blinding of participants and personnel, blinding of outcome assessment, incomplete outcome data, selective reporting and other bias. Each domain was measured as “high risk”, “low risk”, or “unclear risk”. Namely, items with sufficient and correct information were judgment was “low risk”, and items that were reported incorrectly were judgment as “high risk”. If the information of the item was insufficient or unsanctioned, they were judged as “unclear risk”. An “unclear risk” judgement was applied if the item was reported, but the risk of bias was unknown. Discrepancies were resolved by a senior reviewer (DBP).

### Statistical analysis

RR with 95 % CI was used as a common measure of the difference in the efficacy of dexamethasone and ondansetron for prophylaxis of postoperative nausea and vomiting across studies. The power analyses of the individual studies (Power and Precision V4) and meta-analysis were all conducted by Review Manager 5.3. I^2^ was used to estimate statistical heterogeneity. When I^2^ <50 %, heterogeneity was considered present, and the fixed-effects model was applied. Otherwise, the randomized-effects model and sensitivity analysis were applied. Publication bias was evaluated by Egger’s test. A *P* value <0.05 was considered statistically significant.

The quality of evidence was evaluated by two reviewers (HWD and AGZ) using the GRADE system (GRADEprofiler 3.6.1). RCTs were considered to have high-quality evidence. However, this assessment could be downgraded for five reasons: risk of bias, inconsistency, indirectness, imprecision and publication bias. Finally, there were four levels of evidence quality, namely high, moderate, low and very low.

## Results

### Identification of eligible studies

**Fig. 1:**
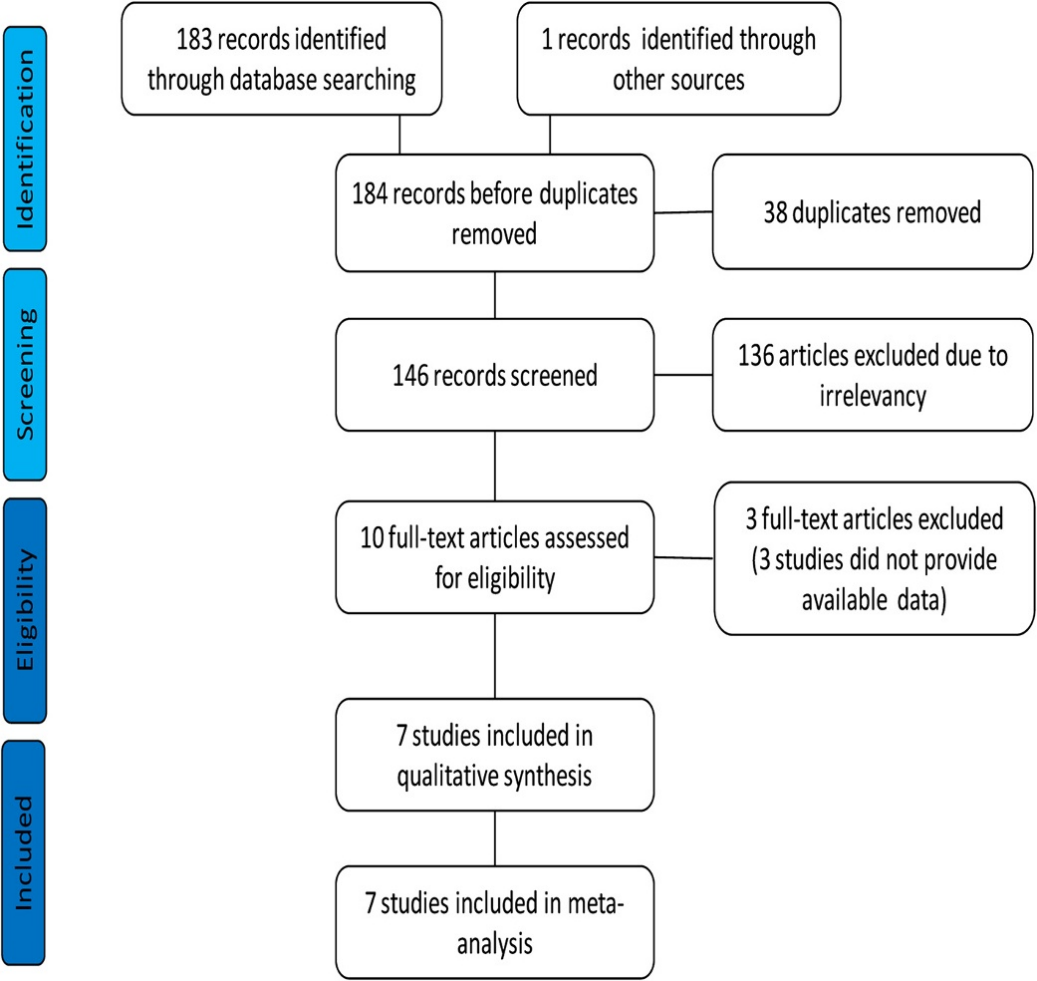
Flow diagram of search strategy and study selection

A total of 184 potentially relevant abstracts were identified. After duplicates were removed, one hundred and forty six unique abstracts remained. After reviewing the abstracts, only ten publications met the inclusion criteria. Three of them were excluded because of incomplete data. Finally, the remaining seven studies [1, 13–18] were included in the present meta-analysis. The search strategy and study selection are presented in the flow diagram of Fig. 1.

### Study characteristics

The characteristics of all included studies are shown in Table 1. All studies were published between 2003 and 2013. The sample sizes of the studies ranged from 40 to 160 (total 608). Drug preparations were administered intravenously before surgery. All included studies were randomized controlled trials and all reported PONV and the rescue anti-emetics used. No significant side effects of dexamethasone or ondansetron were observed in these studies. The definition of PONV was only clearly stated in two studies (Gautan B et al. and Ashwani khimar et al.).

**Table 1:** Characteristics of trials included in systematic review

Study	No. of patients (dexamethasone/ondansetron)	Patient characteristics	Jadad score	Type of anaesthesia	Intra-abdominal pressure
D’souza N et al.	61(30/31)	Adult patients undergoing laparoscopic gynaecologic surgery	4	General	10-14 mmHg
Ashwani K et al.	160(80/80)	Adult patients undergoing laparoscopic cholecystectomy	4	General	None
Erhan Y et al.	40(20/20)	Adult patients undergoing laparoscopic cholecystectomy	4	General	12 mmHg
Gautan B et al.	95(47/48)	Adult patients undergoing laparoscopic cholecystectomy	5	General	Below 15 mmHg
Aighanem SM et al.	120(60/60)	Adult patients undergoing laparoscopic cholecystectomy	5	General	10-16 cm of water
Mohammad E et al.	92(46/46)	Adult patients undergoing laparoscopic cholecystectomy	5	General	Below 15 mmHg
Yuksek MS et al.	40(20/20)	Adult patients undergoing laparoscopic gynaecologic surgery	4	Combined general epidural anaesthesia	12-18 mmHg

### Meta-analyses of primary outcomes

#### Postoperative nausea and vomiting (0–24 h)

**Fig. 2:**
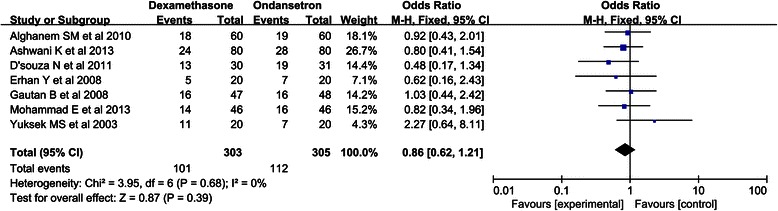
Forest plot of PONV within 24 h of surgery

The results are shown in Fig. 2 and Table 1. PONV within 24 h was studied in seven trials [1, 13–18]. Overall, the rates of PONV in the dexamethasone and ondansetron groups were 33.3 and 36.7 %, respectively. Dexamethasone was not associated with a significant reduction in the incidence of PONV (RR, 0.91; 95 % CI, 0.73-1.13; *P* = 0.39), but no evidence of heterogeneity was observed among the remaining studies (I^2^ = 0 %; *P* = 0.71).

### Postoperative nausea and vomiting at different stages

**Fig. 3:**
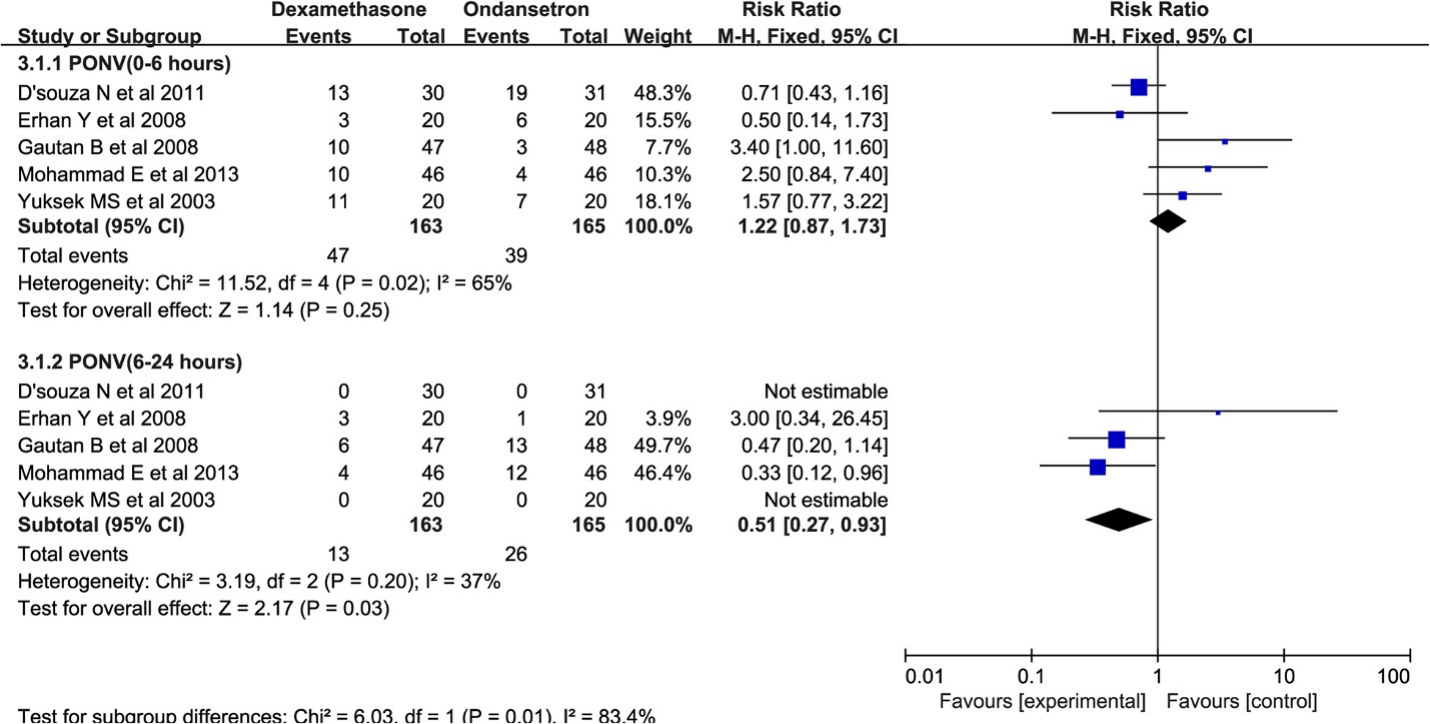
Forest plot of PONV at different stages

**Fig. 4:**
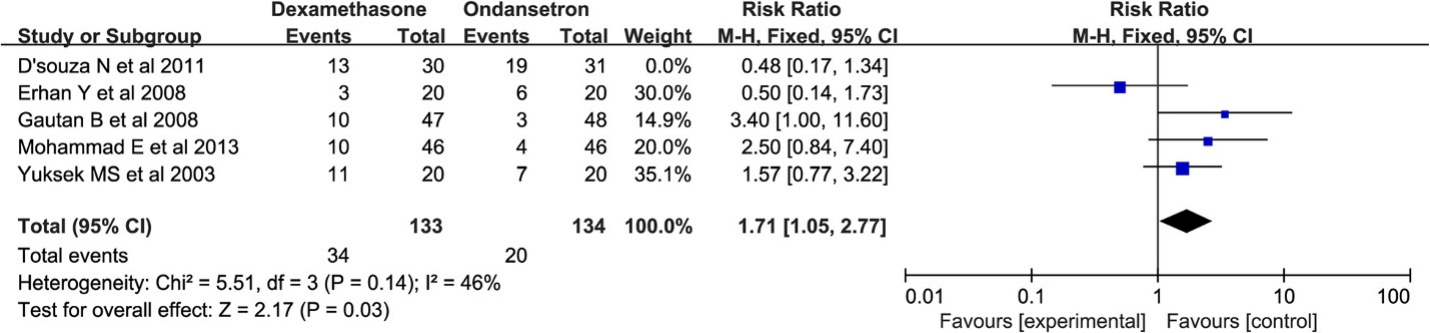
Forest plot of Sensitivity Analysis in the early postoperative stage (0–6 h)

Furthermore, we conducted a subgroup meta-analysis based on the postoperative stage to explore the efficacy of dexamethasone for the prevention of PONV compared with that of ondansetron. Dexamethasone was not superior to ondansetron in preventing PONV in the early postoperative stage (0–6 h) (RR, 1.22, 95 % CI 0.87-1.73; *P* = 0.25), but there was significant heterogeneity in these data among the studies (I^2^ = 65 %; *P* = 0.02). After the exclusion of two trials that did not have any occurrence of PONV in the late postoperative stage (6–24 h), dexamethasone was found to be superior to ondansetron in preventing PONV in the late postoperative stage (RR, 0.51, 95 % CI, 0.27-0.93; *P* = 0.03), and the heterogeneity among the studies was more moderate for these data than for the data for the early postoperative stage (I^2^ = 37 %; *P* = 0.20) (Fig. 3). Subsequently, a sensitivity analysis was carried out to explore the potential source of heterogeneity in the early postoperative stage. As shown in Fig. 4, the results of Nita D’souza et al. [1] were completely out of the range of those of the other studies and this probably contributed to the observed heterogeneity. After excluding this study, the results suggested that compared with dexamethasone, ondansetron was associated with a decreased of incidence PONV (RR 1.71, 95 % CI, 1.05-2.77; *P* = 0.03).

### Meta-analyses of secondary outcomes (0–24 h)

**Fig. 5:**
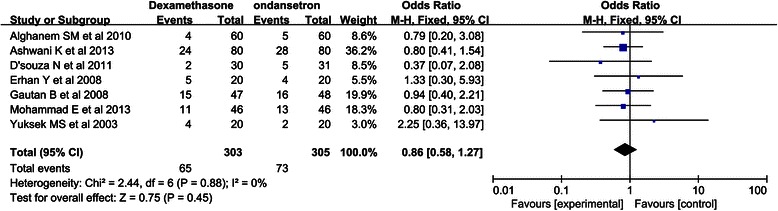
Forest plot of anti-emetics used within 24 h postoperatively

Figure 5 describes the postoperative anti-emetics used within 24 h. The results of these studies suggested no difference in the overall postoperative anti-emetics between the dexamethasone and ondansetron groups (RR, 0.90, 95 % CI, 0.67-1.19; *P* = 0.45). Additionally, no heterogeneity in any of the secondary outcomes was observed (I^2^ = 0 %; *P* = 0.88).

### Quality assessment and quality of evidence

**Fig. 6:**
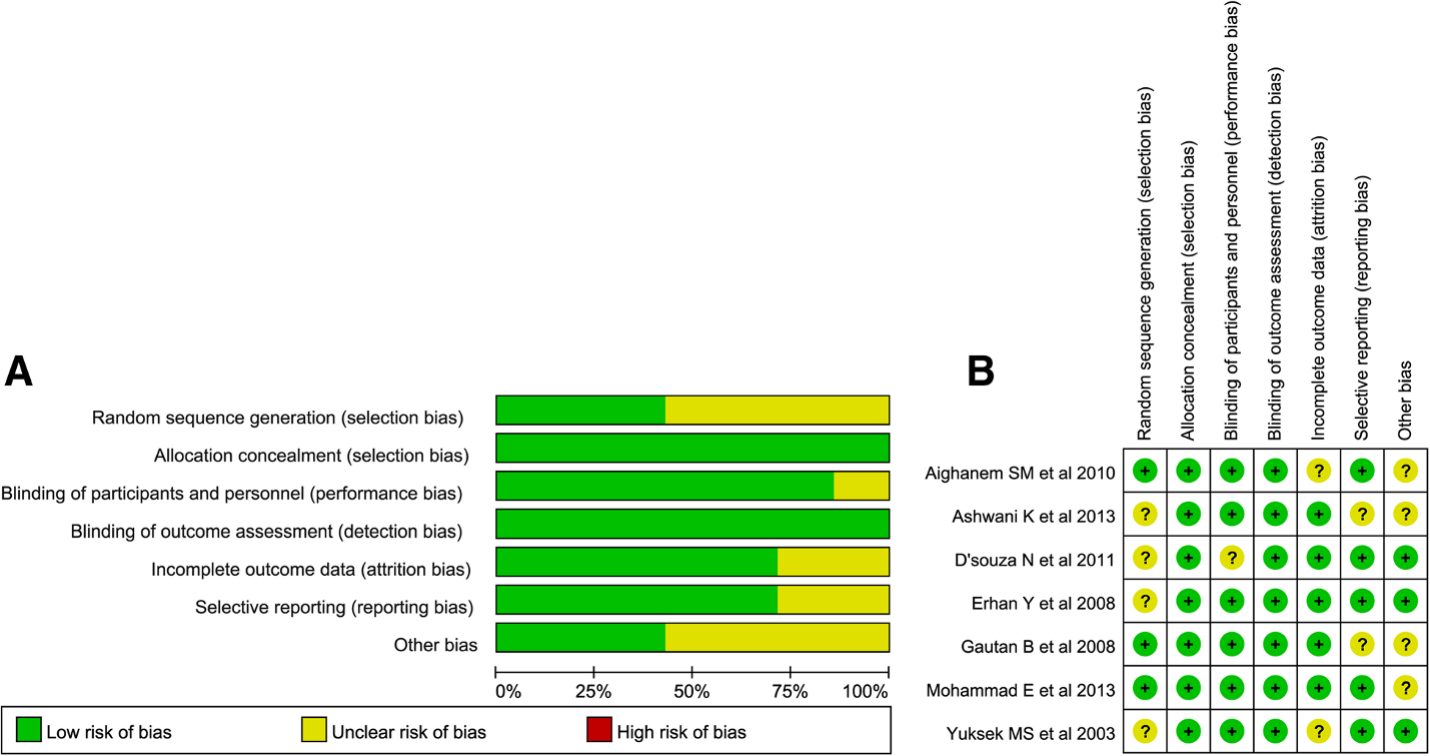
**a** Risk of bias grap. **b** Risk of bias summary

This systematic review included 7 RCTs: the baseline characteristics of patients were reported by all trials. Only one trial mentioned the method of randomization (Fig. 6).

This meta-analysis examined four outcomes: PONV (0–24 h, 0–6 h and 6–24 h) and postoperative anti-emetics (0–24 h). The quality of the evidence for each outcome is presented in Table 2.

**Table 2:** GRADE evidence profile

No. of patients	Effect					Quality				
NO. of studies	Risk of bias	Inconsistency	Indirectness	Imprecision	Other considerations	Dexamethasone	Ondansetron	Relative(95 % CI)	Absolute	
7	No serious risk of bias	No serious inconsistency	No serious indirectness	Serious	None	101/303(33.3 %)	112/305(36.7 %)	RR 0.91(0.72-1.12)	33 fewer per 1000 (from 103 fewer to 44 more)	MODERATE
5	No serious risk of bias	No serious inconsistency	No serious indirectness	Serious	None	47/163(28.8 %)	39/165(23.6 %)	RR 1.25(0.84-1.76)	59 more per 1000 (from 38 fewer to 180 more)	MODERATE
5	No serious risk of bias	No serious inconsistency	No serious indirectness	Serious	None	13/163(8 %)	26/165(15.8 %)	RR 0.48(0.25-0.92)	82 fewer per 1000 (from 13 fewer to 118 more)	MODERATE
7	No serious risk of bias	No serious inconsistency	No serious indirectness	Serious	None	65/303(21.5 %)	73/305(23.9 %)	RR 0.89(0.64-1.19)	26 fewer per 1000 (from 86 fewer to 45 more)	MODERATE

### Power analysis

Although statistical results were presented in some studies, a portion of the primary data was unavailable. The available data were reassessed by a power analysis with an α level of 0.05 (Table 3). The power of the individual studies ranged from 5.1 to 64 %. The power of the meta-analysis with respect to PONV (0–24 h, 0–6 h and 6–24 h) and postoperative anti-emetics (0–24 h) was 12.2, 17.9, 64 and 9.1 %, respectively (Table 3).

**Table 3:** Power analysis of the studies

	PONV(0–24 h)	PONV (0–6 h)	PONV (6–24 h)	Anti-emetics used (0–24 h)
Ashwani K et al.	10.30 %	NA	NA	10.30 %
Gautan B et al.	5.10 %	57 %	45.30 %	5.50 %
Mohammad E et al.	6.90 %	42.40 %	64 %	8.50 %
Yuksek MS et al.	24.20 %	24.20 %	NA	14.10 %
D’souza N et al.	28.70 %	28.70 %	NA	23.60 %
Alghanem SM et al.	5.20 %	NA	NA	7.10 %
Erhan Y et al.	10.50 %	20.20 %	18 %	6.60 %
All studies	12.20 %	17.90 %	64 %	9.10 %

*NA* Not available

### Publication bias

Egger^’^s test did not reveal any significant difference with respect to all outcomes. This result indicated that no publication bias existed (t = 0.66, *P* = 0.537).

## Discussion

This is the first meta-analysis to compare the efficacy of dexamethasone with that of ondansetron in preventing PONV after laparoscopic surgery. The pooled meta-analysis of 7 RCTs using a fixed-effects model suggested that there were no significant differences between dexamethasone and ondansetron in regards to the incidence of PONV or postoperative anti-emetics used during the first 24 h after laparoscopic surgery. Ondansetron was more effective at decreasing PONV in the early postoperative stage (0–6 h), while dexamethasone was more effective at decreasing PONV in the late postoperative stage (6–24 h).

Glucocorticoids bind to intracellular glucocorticoid receptors, and exert their effects via gene transcription [19]. As changes to both gene expression and protein synthesis take time, most effects of corticosteroids are not instantaneous, rather, they only become apparent after several hours. Therefore, glucocorticoids usually take 1–2 h to have biologic effects, and this also depends on the route of administration [20]. This may explain why dexamethasone was found to significantly decrease PONV in the late postoperative stage (6–24 h) rather than in the early postoperative stage (0–6 h) in our data analysis. Interestingly, Thomas and Jones [21] found a failure of prophylaxis during the first 3 h after laparoscopic surgery in 28.3 % of patients who had received dexamethasone compared to with 22 % of patients who had received ondansetron. The late onset and prolonged antiemetic efficacy of dexamethasone may be attributed to its prolonged biological half-life (36–72 h) [22]. Accordingly, the timing of dexamethasone administration is important. It is necessary to administrate dexamethasone 1–2 h preoperatively [23], especially for short surgical procedures. The findings of this meta-analysis depend on the quality of the included primary trials. Despite the declaration of randomization design, only one trial reported sequence generation, which might decrease the level of evidence of this meta-analysis.

Based on the GRADE system, the qualities of outcomes were all “moderate”, and evidence quality was degraded owing to this imprecision. Only two studies clearly defined PONV.

To assess the probability of correctly rejecting the null hypothesis, a power analysis was performed. If H_1_ is true, the power is 1-β. To guard against Type I error, α is typically set to 0.05, and to guard against Type II error, β is set to 0.20 [24–26]. Thus, a power of more than 80 % is necessary to reject the null hypothesis. In our meta-analysis, the power was <80 %. Therefore, this meta-analysis did not provide enough evidence on the effects of the study drugs and more high level studies are required.

In our meta-analysis, the patients enrolled were quite homogeneous. The studies all had Jadad scores of ≥ 4 and were of high quality. For all studies, a fixed-effects model and test of heterogeneity between trials resulted in an I^2^ value of (0.0 %) and a *P* of value (0.71), indicating no heterogeneity. The participants in all studies were well matched (e.g., sex, age, ASA grade, administration time, method of surgery/anaesthesia, et al.). However, several limitations of this meta-analysis should be taken into account. First, there was no gold standard for the definition of PONV, resulting in possible overestimation or underestimation of the true effect of dexamethasone administration compared with that of ondansetron. Furthermore, this meta-analysis was based on studies published in the English language, which may have generated bias. Next, the sample sizes of the studied individual trials were small or moderate. The over difference in the incidence of PONV (0–24 h) was not different between the dexamethasone and ondansetron groups, which may be due to the small sample size and lack of evidence. Finally, we selected published studies, and many studies were not registered on clinical trial data-bases. Data from unpublished literature could be missing, which would lead to bias. However, the Egger’s test result suggested that no publication bias existed.

Nonetheless, our study provides useful evidence for future studies on PONV. Different drugs (dexamethasone and ondansetron) have different working times and half-lives, so attention should be paid to the time-effect relationship of these drugs. Further studies may also focus on the safety of dexamethasone, as long-term corticosteroid administration causes side effects such as wound healing delays, infection, and adrenal suppression. Moreover, in our study, the drugs were only compared when they were used in patients undergoing laparoscopic surgery, so other medical situations in which these drugs are used should also be studied.

## Conclusion

In summary, dexamethasone was equally effective and as safe as ondansetron in preventing PONV. However, in the late postoperative stage (6–24 h), dexamethasone probably has an advantage over ondansetron. Considering the limitations of this study, our findings should be considered with caution, and large-scale studies are needed to confirm our findings.

## Abbreviations

RR: Risk ratios
PONV: Postoperative nausea and vomiting
PICOS: Patient, intervention, comparison, outcome and study design
ASA: American Society of Anesthesiology
RCTs: Randomized controlled trials
